# Comparative Analysis of the Potential for Germ Cell (GC) Differentiation of Bovine Peripheral Blood Derived-Mesenchymal Stem Cells (PB-MSC) and Spermatogonial Stem Cells (SSC) in Co-Culture System with Sertoli Cells (SC)

**DOI:** 10.3390/ani13020318

**Published:** 2023-01-16

**Authors:** Moisés N. Segunda, Carlos Díaz, Cristian G. Torres, Víctor H. Parraguez, Mónica De los Reyes, Oscar A. Peralta

**Affiliations:** 1 Department of Animal Production Sciences, Faculty of Veterinary and Animal Sciences, University of Chile, Santa Rosa 11735, Santiago 8820808, Chile; 2 Doctorate Program of Forestry, Agriculture, and Veterinary Sciences (DCSAV), University of Chile, Santa Rosa 11315, Santiago 8820808, Chile; 3 Faculdade de Medicina Veterinária, Universidade José Eduardo dos Santos, Bairro Santo António-Avenida Nuno Alvarez, Huambo 555, Angola; 4 Doctorate Program in Sciences, UNED, Bravo Murillo 38, 28015 Madrid, Spain; 5 Department of Clinical Sciences, Faculty of Veterinary and Animal Sciences, University of Chile, Santa Rosa 11735, Santiago 8820808, Chile; 6 Department of Biological Sciences, Faculty of Veterinary and Animal Sciences, University of Chile, Santa Rosa 11735, Santiago 8820808, Chile

**Keywords:** mesenchymal stem cells, MSC, spermatogonial stem cells, SSC, Sertoli cells, SC, germ cell (GC) differentiation

## Abstract

**Simple Summary:**

The primary function of spermatogonial stem cells (SSC) in the testis is to produce germ cells (GC) and sperm. This function may be recreated under laboratory conditions using in vitro three-dimensional (3D) cell culture systems. This technology may be used for the treatment of men infertility and in animals for dissemination of elite genetics and conservation of endangered species; however, SSC are scarce and difficult to culture. Potentially, mesenchymal stem cells (MSC) derived from peripheral blood (PB-MSC) may also be candidates for GC production, due to their simplicity for culture and differentiation capacity. The aim of the present study was to compare the potential for production of GC of bull PB-MSC and SSC using an in vitro 3D co-culture system with Sertoli cells (SC). Samples of PB-MSC or SSC co-cultures with SC were collected on days 0, 7, 14 and 21 and analyzed for pluripotency, GC and mesenchymal gene expression. Co-culture of bull PB-MSC or SSC+SC in 3D resulted in differential expression of genes associated to pluripotency and GC differentiation. In conclusion, profiles of expression of pluripotency and GC genes indicate that SSC display a more robust GC differentiation profile compared to PB-MSC, when co-cultured in a 3D system with SC.

**Abstract:**

Although spermatogonial stem cells (SSC) constitute primary candidates for in vitro germ cell (GC) derivation, they are scarce and difficult to maintain in an undifferentiated state. Alternatively, mesenchymal stem cells (MSC) are also candidates for GC derivation due to their simplicity for culture and multipotential for transdifferentiation. The aim of the present study was to compare the GC differentiation potentials of bull peripheral blood-derived MSC (PB-MSC) and SSC using an in vitro 3D co-culture system with Sertoli cells (SC). Samples of PB-MSC or SSC co-cultures with SC were collected on days 0, 7, 14 and 21 and analyzed for pluripotency, GC and mesenchymal marker expression. Co-culture of PB-MSC+SC resulted in down-regulation of *NANOG* and up-regulation of *OCT4* at day 7. In comparison, co-culture of SSC+SC resulted in consistent expression of *NANOG*, *OCT4* and *SOX2* at day 14. During co-culture, SSC+SC increased the expression of *DAZL*, *PIWIL2*, *FRAGILIS* and *STELLA* and activated the expression of *STRA8*, whereas co-culture of PB-MSC+SC only increased the expression of *DAZL* and *PIWIL2*. Thus, co-culture of bull PB-MSC+SC and SSC+SC in 3D SACS results in differential expression of pluripotency and GC markers, where bull SSC display a more robust GC differentiation profile compared to PB-MSC.

## 1. Introduction

Mesenchymal stem cells (MSC) represent a novel source of multipotent progenitor cells capable of differentiating into germ layer-derived cell lineages through the process of transdifferentiation [1]. MSC display as a fundamental characteristic in culture, the ability to differentiate towards mesodermal lineages including osteocytes, chondrocytes and adipocytes [2,3]. In addition, it has been reported that MSC can differentiate under in vitro conditions into cell lineages derived from endodermal and ectodermal germ layers, including hepatocytes and neurons, respectively [4,5,6]. These features suggest that the in vitro differentiation potential of MSC may be greater than initially reported.

MSC are widely distributed and can be isolated from various animal tissue sources including adipose tissue, bone marrow, umbilical cord, placenta and peripheral blood [3,7,8]. Although MSC from different tissue sources share similar biological characteristics, peripheral blood-derived MSC (PB-MSC) possess some collection advantages since they can be obtained with minimal invasion compared with bone marrow-derived MSC (BM-MSC) and adipose tissue-derived MSC (AT-MSC), which require invasive and painful procedures [9]. Moreover, a continuous source of PB-MSC may be obtained from males and females compared to umbilical cord (UC-MSC) or placenta (PL-MSC), which can only be collected after gestation. Despite these advantages, the reduced proportion of PB-MSC in the steady-state blood may create some challenges in terms of the yield of cells collected [9,10].

Spermatogonial stem cells (SSC) can be differentiated into meiotic germ cells (GC) in vitro, which have suggested the potential use of SSC for the treatment of male human infertility, especially in azoospermic or oligospermic men [11]. GC derivation from SSC may also have potential application in animal reproduction, as an alternative method for dissemination of elite genetics and conservation of endangered species [12]. Despite its potential applications, bovine SSC are scarce in the testicular tissue and are difficult to identify and maintain in an undifferentiated state under in vitro conditions [13]. As an alternative, MSC hold the promise as a stem cell donor for GC differentiation and transplantation. Some of the potential features of MSC for GC derivation include their abundant tissue sources, simplicity for isolation and culture, ease of in vitro expansion, presence of specific markers, potential for transdifferentiation towards GC, immune evasive potential, and migratory and colonization capacity [14,15]. Using different protocols, our group and others have reported that MSC can acquire the phenotype of early GC in culture, when exposed to factors such as retinoic acid (RA), bone morphogenetic protein 4 (BMP4) and transforming growth factor β1 (transforming growth factor β1; TGF-β1) [1,16]. Recently, the potential of MSC to reach early GC states has also been evaluated through the overexpression of GC genes *DAZL*, *STRA8* and *BOULE* [17]. Furthermore, an alternative differentiation strategy has consisted of co-culturing MSC with Sertoli cells (SC), which has resulted in increased expression of GC markers including *DAZL* and *PIWIL2* [18,19,20]. These studies have generated promising results on the potential of bovine MSC for differentiation into GC and, in addition with other features, suggest that MSC may be adequate candidates for derivation of GC.

Germinal differentiation and spermatogenesis in vivo depend on the surrounding microenvironment and interaction with other testicular cell types including SC [21]. These cells regulate the cycle of GC by secreting factors necessary for their viability, proliferation and differentiation, including BMP4, TGF-β1 and AR [18]. In addition, SC form tight junctions that structure the blood–testis barrier, which acts as a filter that selects the plasmatic compounds useful for spermatogenesis and prevents the diffusion of self-antigens from the interior of the seminiferous tubule into the blood [11,18]. This barrier also influences the chemical composition of the fluid in the lumen, which contains high levels of androgens, estrogens, potassium, inositol, glutamic and aspartic acid, essential for the proliferation and differentiation of GC [22,23,24]. Thus, the proliferation and differentiation of SSC in vivo are influenced by the junctions and the spatial arrangement of SC. Moreover, various studies have emphasized the importance of cell interaction in differentiation systems [11,25,26]. This interaction allows an organized structure and function and the participation of cell-specific adhesion molecules, their association with the extracellular matrix, the cytoskeleton and the metabolic state, which together respond to the stimuli in the extracellular medium [11].

Since SSC represent the standard for differentiation towards GC and spermatogenesis, the differentiation potential of PB-MSC may be compared with SSC in order to better understand their potential of GC differentiation. The expression patterns of differentiation biomarkers in PB-MSC and SSC would allow us to understand the possible mechanisms that control and limit their GC differentiation capacity under in vitro conditions. Thus, the aim of the present study was to compare the differentiation potentials of bull PB-MSC and SSC into GC using an in vitro 3D co-culture system with SC.

## 2. Materials and Methods

### 2.1. Ethics

All experimental procedures were previously approved by the Institutional Committee for Care and Use of Animals (CICUA) at the University of Chile (certificate 19266-VET-UCH) and the Biosafety Committee of the Faculty of Veterinary and Animal Sciences from the University of Chile (certificate 140).

### 2.2. Experimental Design

PB-MSC were collected and polled from three Angus beef bulls (age 1–3 years) belonging to the Faculty of Veterinary and Animal Sciences from the University of Chile. Each pool represented a biological replicate and analyses, and experiments were performed thrice. Pooling tissue samples was performed in an effort to reduce individual biological variation. SC and SSC were isolated from adult bull testis (n = 10) derived from a local abattoir. All cell types were characterized accordingly to the gene expression of specific markers for MSC *(+CD73*, *+CD105*, -*CD34* and -*CD45*), SSC (+*UCHL1* and +*CD90*) and SC (+*WT1* and +*RA*) using quantitative PCR (QPCR). Cells were also characterized for the protein expression of markers for MSC (*CD73*), SSC (*UCHL1*) and SC (*WT1*) using immunofluorescence. PB-MSC and SSC (2.5 ×10^4^ cells/cm^2^) were co-cultured with SC (2.5 ×10^4^ cells/cm^2^) in 3D soft agar co-culture system (SACS). PB-MSC and SSC were considered target cells and SC were used as effector cells. Experimental controls corresponded to PB-MSC, SSC and SC (2.5 ×10^4^ cells/cm^2^) and were cultured separately. Samples from each co-culture and single culture were collected on days 0, 7, 14 and 21. GC differentiation of PB-MSC and SSC was determined by quantifying the gene expression of the pluripotency markers *OCT4*, *NANOG* and *SOX2*; GC genes *FRAGILIS*, *STELLA*, *DAZL*, *PIWIL2*, and *STRA8* by Q-PCR; and *OCT4*, *NANOG*, *DAZL*, *PIWIL2* and *STRA8* by immunofluorescence

### 2.3. Isolation, Purification, and Characterization of Bull PB-MSC

Blood samples (25 mL per animal) were collected from the coccygeal vein of 1- to 3-year-old bulls using vacutainer tubes containing heparin. Blood samples were kept at 4 °C and transported to the laboratory within 1 h after collection. PB-MSC were isolated according to the previously reported protocol [7]. Briefly, blood samples were diluted 1:1 in phosphate buffered saline (PBS; pH: 7.4) and layered onto 20 mL of 1.076 mM Percoll solution (Biochrom AG, Berlin, Germany). Subsequently, the samples were centrifuged at 1600× *g* for 20 min. The mononuclear cell interface was recovered, subsequently washed in PBS and resuspended in MSC culture medium containing low glucose DME-F12 (Dulbecco’s Modified Eagle F12) medium, supplemented with 10% fetal bovine serum (FBS), 100 μg/mL of amphotericin B, 100 μg/mL of streptomycin, and 100 IU/mL of penicillin (Gibco, Life Technologies, Carlsbad, CA, USA). The cells obtained from each 25 mL of blood were cultured in T25 culture bottles (Corning, Toronto, Canada) in MSC culture medium and incubated in an atmosphere of humidified air with 5% CO_2_ and 38 °C. Non-adherent cells were discarded after 48 h by changing the culture medium. Isolated cell colonies were maintained in MSC medium until reaching ~75% confluency. Cells were then detached with 0.5% trypsin-EDTA (Invitrogen, BRL, Burlington, Canada) for subculture and used in subsequent experiments. The expression of MSC cell surface markers, both mesenchymal (+*CD73* and +*CD105*) and hematopoietic (-*CD34* and -*CD45*), were evaluated by Q-PCR, and *CD73* by immunofluorescence, to characterize the cell phenotype established by the International Society for Cell Therapy (ISCT; [2]).

### 2.4. Isolation, Purification, and Characterization of Bull SC

SC were isolated from adult bull testicular tissue by sequential enzymatic digestion according to a modified protocol [19]. Testicular tissue was mechanically dissociated using scissors and forceps. Subsequently, an enzymatic digestion of the tissue was performed in DME-F12 medium with L-glutamine supplemented with 2 mg/mL of collagenase type I (Sigma-Aldrich, St Louis, MI, USA) and 2 mg/mL of DNase I (Sigma Aldrich) for 45 min. The digestion reaction was blocked by adding SC culture medium composed of DME-F12 supplemented with 10% FBS, 100 IU/mL penicillin, 100 μg/mL streptomycin and 100 μg/mL of amphotericin B. Then, the cell suspension was centrifuged at 500× *g* for 10 min at 4 °C and the pellet was filtered through a 100 μm mesh (Corning, Canada, USA). Cells were aliquoted and seeded in T25 bottles with SC culture medium. SC cultures were subsequently incubated at 38 °C in a humidified atmosphere with 5% CO_2_. After one hour, the supernatant containing GC was removed and the cells adherent to the plastic were washed twice with PBS and subsequently cultured in fresh medium. To obtain SC cultures with a ratio of 80–90%, the cultures were hypotonically treated after 48 h, using 20 mM Tris-HCl (pH 7.4) for 2.5 min to lyse residual GC. Subsequently, the plates were washed twice with PBS, and, after changing the medium, the cells were cultivated in an atmosphere with 5% CO_2_ at 38 °C. When adherent cells reached 80% to 90% confluency, they were detached using 0.5% trypsin-EDTA and transferred to new plates for expansion and removal of residual GC. During cultivation, non-adherent GC were removed by repeated washing. The morphological features of SC were examined and photographed using phase contrast microscopy. A testicular extract (TE) sample was used as a positive control and was prepared by mechanical maceration of testicular tissue pieces in lysis buffer (Zymo Research, Irvine, CA, USA) for RNA extraction. The expression of SC markers *WT1* and *AR* was evaluated by Q-PCR and *WT1* by immunofluorescence.

### 2.5. Isolation, Purification, and Characterization of Bull SSC

SSC were isolated from adult testicular tissue by a modified adhesion separation protocol previously described [27]. After testicular decapsulation, the tissue was mechanically dissociated by a two-step mechanical and enzymatic digestion. First, the testes were sectioned into small pieces and incubated in the enzyme solution that included DME-F12 supplemented with 0.5 mg/mL collagenase type I (Sigma-Aldrich), 0.5 mg/mL trypsin-EDTA (Invitrogen, BRL, Canada) and 0.5 mg/mL DNase I (Sigma Aldrich) for approximately 45 min with shaking and pipetting at 38 °C. The digested testicular tissues were washed in DMEM, and, after precipitation, the supernatant containing interstitial cells from the seminiferous tubules was discarded. The remaining tissue was digested during the second stage of enzymatic digestion until separation of its constituent cells. The cell suspension was two-step filtrated through 100-µm and 40-µm filters, and then the cell suspension was cultured twice for 24 h in SC culture medium. Separation of SSC (non-adherent cells) from SC (adherent cells) was performed by differential plate method during 24 h of incubation. For this, the obtained testicular cells were transferred to new culture bottles and cultured overnight in SC culture medium at 38 °C in 5% CO_2_. After this step, the floating cells were collected and centrifuged at 1800× *g* for 5 min and were characterized by expression of specific biomarkers including *UCHL1* and *CD90* [28] using Q-PCR and *UCHL1* using immunofluorescence techniques.

### 2.6. Co-Culture of PB-MSC or SSC with SC in 3D SACS

The PB-MSC or SSC co-culture with SC was based on the 3D SACS culture system according to the previously modified protocol [29]. The 3D SACS consisted of 2 phases: (1) the gel phase or soft top layer with 0.35% (*w*/*v*) agarose, and (2) the solid phase or bottom layer with 0.5% (*w*/*v*) agarose. For this, 0.7% (*w*/*v*) agarose and 1% (*w*/*v*) agarose were dissolved in distilled water, and an equal volume of culture medium was added to these solutions to obtain a concentration of 0.35% and 0.5%. To establish the solid bottom layer (800 μL final volume), 2 h before cell culture, 400 μL of 1% agarose solution was mixed with 400 μL of SC culture medium and transferred into a 12-well plate. The plates were refrigerated at 4 °C until the agarose in this layer solidified. To establish the upper layer (final volume of 200 μL), 2.5 ×10^4^ cells/cm^2^ of MSC or SSC were mixed with 2.5 × 10^4^ cells/cm^2^ SC in 100 μL of DME-F12 supplemented with 10% FBS, and mixed with 100 μL of 0.7% agarose at 38 °C. The prepared cell suspension was then pipetted onto the solid layer. All plates were kept at 38 °C and 5% CO_2_ for 3 weeks, and culture media were replaced every 3 days. Cells were collected by adding 1 mL of PBS to each well and centrifuging each cell suspension at 2000× *g* for 5 min. Cell pellets were fixed in lysis buffer or 4% paraformaldehyde for Q-PCR or immunofluorescence analyses, respectively.

### 2.7. Q-PCR Analysis

The quantification of the mRNA levels of the housekeeping genes *GAPDH* and *β-ACTIN*, pluripotency genes *OCT4* and *NANOG*, GC markers *FRAGILIS*, *PIWIL2* and *STELLA*, SSC markers *UCHL1* and *CD90* and male GC markers *DAZL* and *STRA8*, was determined using Q-PCR (Table 1). Total RNA was isolated from cells using a Quick-RNA MiniPrep kit (Zymo Research) following the manufacturer’s instructions. Total RNA was quantified using a Qubit 3.0 (Invitrogen, Fluorometer, CA, USA). Genomic DNA digestion was performed using DNase I from the Quick-RNA MiniPrep kit (Zymo Research) following the manufacturer’s instructions. The cDNA was synthesized and amplified using an Affinity Script Q-PCR cDNA Synthesis Kit (Agilent Technologies, Santa Clara, CA, USA), using a Step One thermocycler (Applied Biosystems, Foster City, CA, USA). The PCR reaction was performed using a Brilliant SYBR Green QPCR Master Mix kit (Agilent Technologies) and an Eco Real-Time PCR System thermocycler (Illumina, San Diego, CA, USA). Each reaction tube consists of 5 μL Sybr Green, 1 μL forward primer, 1 μL reverse primer, 2 μL nuclease-free H_2_O and 5 ng cDNA. The cDNA amplification was extended for 40 cycles, and relative expression analysis was performed using the ΔΔCt (Ct: threshold value) method normalized with both *GAPDH* and *β-ACTIN* housekeeping genes [30].

### 2.8. Immunofluorescence Analysis

Protein expression of Sertoli cell (*WT1*), SSC (*UCHL1*), pluripotency (*OCT4* and *NANOG*), MSC (*CD73*) and male GC (*DAZL*, *STRA8*, *STELLA* and *PIWIL2*) markers were immunodetected at day 14 in co-cultures and single cultures. Culture samples were fixed with 4% PFA at 4 °C for 20 min. Subsequently, the samples were washed and blocked with 3% bovine serum albumin (BSA) diluted in PBS (pH: 7.4) for 45 min. Markers were immunodetected using rabbit polyclonal anti-*WT1* (Cat. # ab89901, Abcam, Boston, MA, USA), mouse polyclonal anti-*OCT4* (Cat. # sc5279, Santa Cruz, Dallas, TX, USA), mouse polyclonal anti-*NANOG* (Cat. # sc293121, Santa Cruz), mouse monoclonal anti-*UCHL1*, (Ct.# 480012, Thermo-Fisher, Waltham, MA, USA), rabbit polyclonal anti-*STRA8* (Cat. # ab49602, Abcam), rabbit monoclonal anti-*CD73* (Cat. # ab137595, Abcam), rabbit monoclonal anti-*PIWIL2* (Ct.# ab85084, Abcam) and rabbit polyclonal anti-*DAZL* (Cat. # ab34139, Abcam), mouse polyclonal anti-*STELLA* (Cat. # ab19878, Abcam), diluted in 3% BSA diluted in PBS (pH: 7.4) (*Wt1* 1:200; *OCT4*, *NANOG*, *STRA8*, *CD73*, *PIWIL2* and *DAZL* 1:50, *UCHL1* and *STELLA* 1:100). Incubation with primary antibodies was performed overnight at 4 °C. The next day, samples were washed once with PBS and twice with 3% BSA diluted in PBS (pH: 7.4) and incubated in secondary IgG anti-mouse antibody conjugated with Alexa-fluor 488 (CT.# A11001, Thermo-Fisher) or IgG anti-rabbit antibody conjugated with FITC (Ct.# ab97050, Abcam) diluted in PBS with BSA 3% (1:500) for 1 h. Next, the samples were washed again twice in PBS with 3% BSA (pH: 7.4) and once with distilled water and subsequently mounted in DAPI Fluoroshield medium (Cat. # ab104139, Abcam). Once the protocol was finished, the samples were stored at 4 °C and protected from light. The samples were photographed using an epifluorescence microscope (Olympus, Tokyo, Japan) and a spectral confocal microscope (Nikon, Tokyo, Japan) connected to a digital camera.

### 2.9. Statistical Analysis

The data obtained represented the mean and the standard deviation. The statistical model considered the relative expression of the biomarkers as dependent variables. The independent variables were the days of culture and the type of treatment. A significance value of *p* < 0.05 was used. Significance of the statistical model was analyzed by one-way ANOVA. The differences between means for culture days and treatments were determined using Tukey’s post-test. Infostat 2020 software (Cordoba, Argentina) was used in all statistical analyses.

## 3. Results

### 3.1. Morphology of Bull PB-MSC, SC and SSC

**Figure 1:**
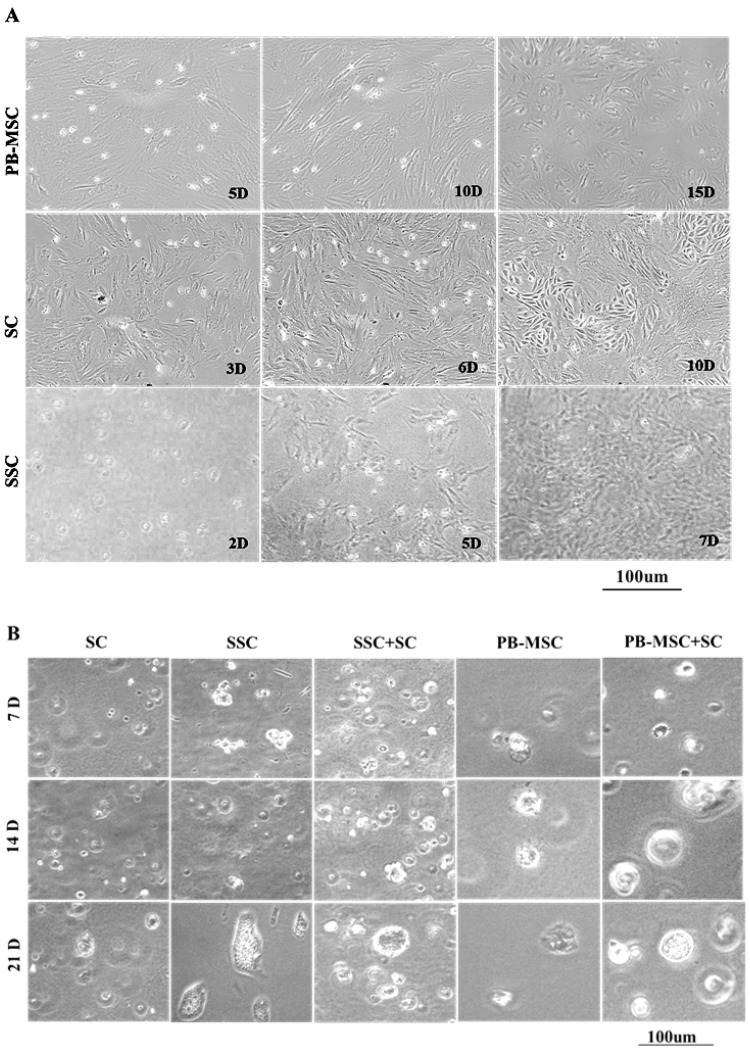
Phase contrast microscopy of single cultures and co-cultures in 3D SACS of bull PB-MSC, SC and SSC. (**A**) PB-MSC presented circular and fibroblastoid morphology at 5D of culture, elongated and heterogeneous morphology at 10D, and circular cells with large nuclei, triangular or circular shape at 15D. Bull SC formed a monolayer of large fibroblastoid cells with heterogenous morphology after removing GC from cultures at 3D of culture, and displayed triangular or circular subpopulations after 1–3 passages at 6D. Finally, distinct SC clusters were observed as cells became more confluent at 10D. Non-adherent SSC with a circular morphology were kept in suspension for 2D, and a population of SSC adherent with an irregular and cuboidal morphology appeared and formed colonies at 5D of culture. SSC proliferated quickly, and then they detached and floated at 7D. (**B**) In 3D SACS, SSC formed large colonies on day 21, PB-MSC and SC lost the elongated fibroblastoid appearance to a circular or cuboidal shape. During culture in 3D SACS, round colonies that increased in size and number could be observed in the SSC+SC and PB-MSC+SC co-cultures.

PB-MSC displayed a diverse morphology that ranged from circular and fibroblastoid at 5 days (5D) of culture to elongated and heterogeneous as they reached confluence at 10 days (10D) after seeding (Figure 1A). Subsequently, subpopulations of circular cells with large nuclei, triangular or circular shape were observed at 15 days (15D) of culture. After removing GC from cultures, bull SC formed a monolayer of large fibroblastoid cells with heterogenous morphology (3D). Similarly, after 1–3 passages (6D), bull SC displayed triangular or circular subpopulations, and distinct cell clusters were observed as cells became more confluent (10D). Non-adherent SSC with a circular morphology were kept in suspension for 2 days (2D), and then a population of adherent cells with an irregular and cuboidal morphology appeared and formed colonies at day 5 of culture (5D); these cells proliferated quickly until day 7, and then they detached and floated (7D).

### 3.2. Morphology of PB-MSC or SSC Co-Cultured with SC in SACS 3D for 21 Days

During culture, SC acquired a circular morphology and retained this morphology until day 21 when they formed confluent colonies (Figure 1B). In general, all adherent cell types, both PB-MSC and SC, changed their morphology and formed round cell colonies. Moreover, SSC were able to remain in culture forming large colonies until day 21 of culture. In co-cultures of SSC+SC and PB-MSC+SC, cells changed their elongated fibroblastoid appearance to a circular or cuboidal morphology that remained until day 21 of culture.

### 3.3. Expression of MSC, SC and SSC Markers in Single Cultures

**Figure 2:**
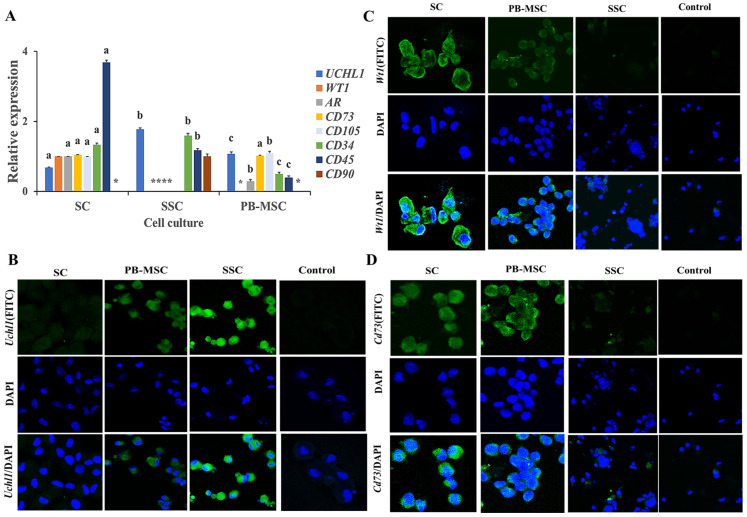
Expression of cell specific markers in single cultures of bull PB-MSC, SSC with SC. (**A**) Gene expression levels of SC markers WT1 and RA were higher (*p* < 0.05) in SC and WT1 and were not detected in SSC or MSC. Gene expression of SSC gene marker UCHL1 was higher (*p* < 0.05) in SSC. *CD90* gene expression was only expressed in SSC. Levels of MSC *CD73* gene marker were similar (*p* > 0.05) between SC and MSC and were not detected in SSC. *CD105* gene marker had higher expression (*p* < 0.05) in MSC compared to SC. *CD45* gene marker had higher expression (*p* < 0.05) in SC comparing with SSC and MSC, whereas *CD34* gene had higher expression (*p* < 0.05) in SSC compared to SC and MSC. (**B**) Immunofluorescence associated to *UCHL1* was observed in SSC. (**C**) Intense, Wt1 immunofluorescence was observed in SC. (**D**) Immunofluorescence associated to *CD73* was observed in MSC. (*) Indicate gene expression was not detected for a single marker. Different superscripts (a, b, c) indicate differences (*p* < 0.05) for the same marker between cell types.

The gene expression of the specific markers of SC, SSC and PB-MSC was determined in all cell types with the aim to determine the purity of the primary cell cultures (Figure 2). Gene expression levels of *UCHL1* were higher (*p* < 0.05) in SSC (1.8 ± 0.014-fold SC) compared to PB-MSC (1.1 ± 0.04-fold SC). Gene expression of *WT1* was detected in SC and was absent in PB-MSC and SSC. Levels of mRNA of *AR* were higher (*p* < 0.05) in SC compared to PB-MSC (0.3 ± 0.02-fold SC) and were not detected in SSC. Gene expression levels of *CD73* were not different (*p* > 0.05) between SC and PB-MSC (1.0 ± 0.03-fold SC) and were not detected in SSC. Levels of mRNA of *CD105* were higher (*p* < 0.05) in PB-MSC (1.17 ± 0.05-fold SC) compared to SC and were not detected in SSC. Gene expression levels of *CD34* were higher (*p* < 0.05) in SSC (1.60 ± 0.04-fold SC) compared to SC and PB-MSC (0.6 ± 0.05-fold SC). Relative gene expression of *CD45* was higher (*p* < 0.05) in SC (3.5 ± 0.03-fold SSC) compared to PB-MSC (0.4 ± 0.04-fold SSC). In comparison, gene expression of *CD90* was only detected in SSC (1.0 ± 0.15). Additionally, a more intense cytoplasmic immunofluorescence pattern of *UCHL1* was detected in SSC compared to PB-MSC (Figure 2B). Moreover, *UCHL1* was not immunodetected in SC. The immunofluorescence associated to *WT1* was observed in SC, was less intense in PB-MSC and was not observed in SSC (Figure 2C). Moreover, immunofluorescence associated to *CD73* was detected in PB-MSC, with less immunofluorescence intensity in SC, and was not observed in SSC (Figure 2D).

### 3.4. Expression of Pluripotency Markers in PB-MSC or SSC Co-Cultured with SC for 21 Days in 3D SACS

**Figure 3:**
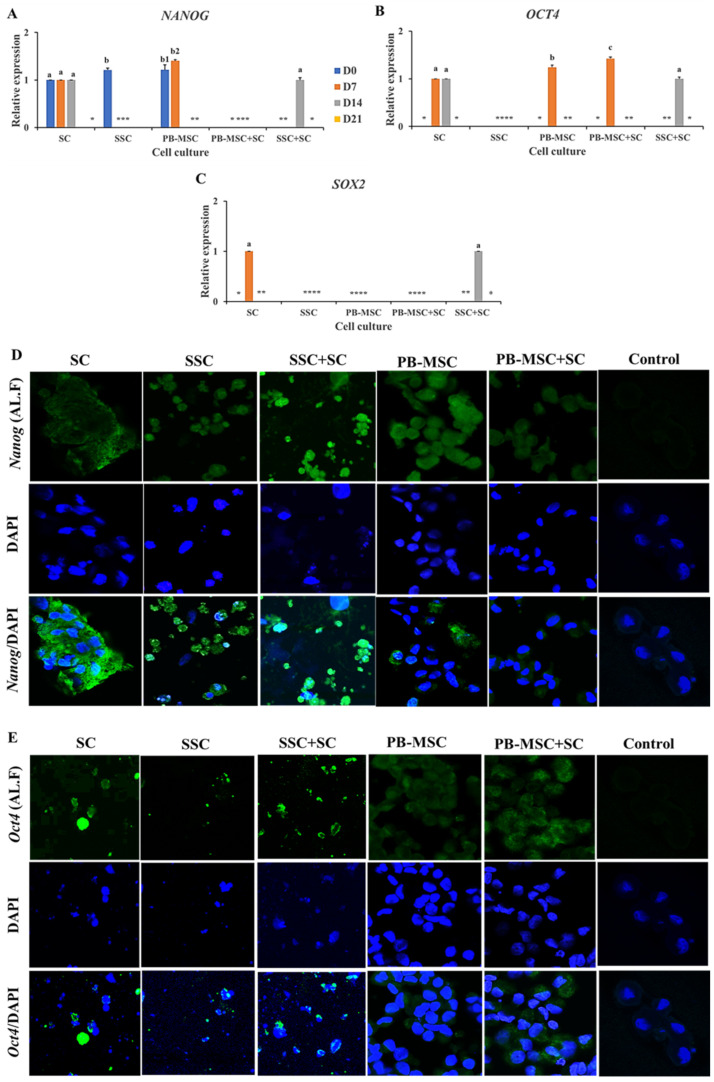
Expression of pluripotency markers *NANOG*, *OCT4* and *SOX2* in single cultures and co-cultures of PB-MSC and SSC with SC in 3D SACS for 21 days. (**A**) Expression levels of *NANOG* were similar (*p* > 0.05) in SSC+SC co-culture compared to SC single culture on day 14. (**B**) Co-culture of PB-MSC+SC up-regulated *OCT4* gene expression on day 7 compared to SC and PB-MSC single cultures. (**C**) *SOX2* gene expression was detected in SC on day 7 and on day 14 in co-cultures of SSC+SC. (**D**) More intense immunofluorescence associated to *NANOG* was observed in SSC+SC co-cultures at day 14. (**E**) Similarly, more intense immunofluorescence associated to *OCT4* was observed in SSC+SC co-cultures at day 14. (*) Indicate gene expression was not detected in a single sampling day. Different superscripts (a, b, c) indicate significant differences (*p* < 0.05) for the same marker between cell types and (1,2) for the same marker between culture days.

Gene expression levels of *NANOG* were similar (*p* > 0.05) in SSC+SC co-culture (1.0 ± 0.03-fold SC) compared to SC single culture on day 14. In comparison, gene expression of *NANOG* were not detected in any days of PB-MSC+SC co-culture, in spite of the fact that mRNA levels of *NANOG* were detected in single cultures of SC and PB-MSC at days 0 and 7 (Figure 3A, Table 2). Accordingly, intense immunofluorescence associated to *NANOG* was observed in SSC+SC co-culture and SC single culture at day 14 (Figure 3D). Co-culture of PB-MSC+SC up-regulated (1.4 ± 0.03-fold SC; *p* < 0.05) *OCT4* gene expression on day 7 (Figure 3B, Table 2) compared to SC and PB-MSC (1.2 ± 0.05-fold SC) single cultures. Levels of *OCT4* gene expression were similar (*p* > 0.05) in co-culture of SSC+SC (1.0 ± 0.03-fold SC) and single culture of SC on day 14. *OCT4* gene expression was not detected on SSC. Immunofluorescence associated to *Oct4* was mainly observed in SSC+SC and MSC+SC co-cultures with a cytoplasmatic and nuclear pattern at day 14 (Figure 3E). *SOX2* gene expression was detected in SC on day 7 and on day 14 in co-cultures of SSC+SC; however, *SOX2* gene expression was not detected in other cell cultures (Figure 3C, Table 2).

### 3.5. Expression of GC Markers in PB-MSC or SSC Co-Cultured with SC for 21 Days in 3D SACS

**Figure 4:**
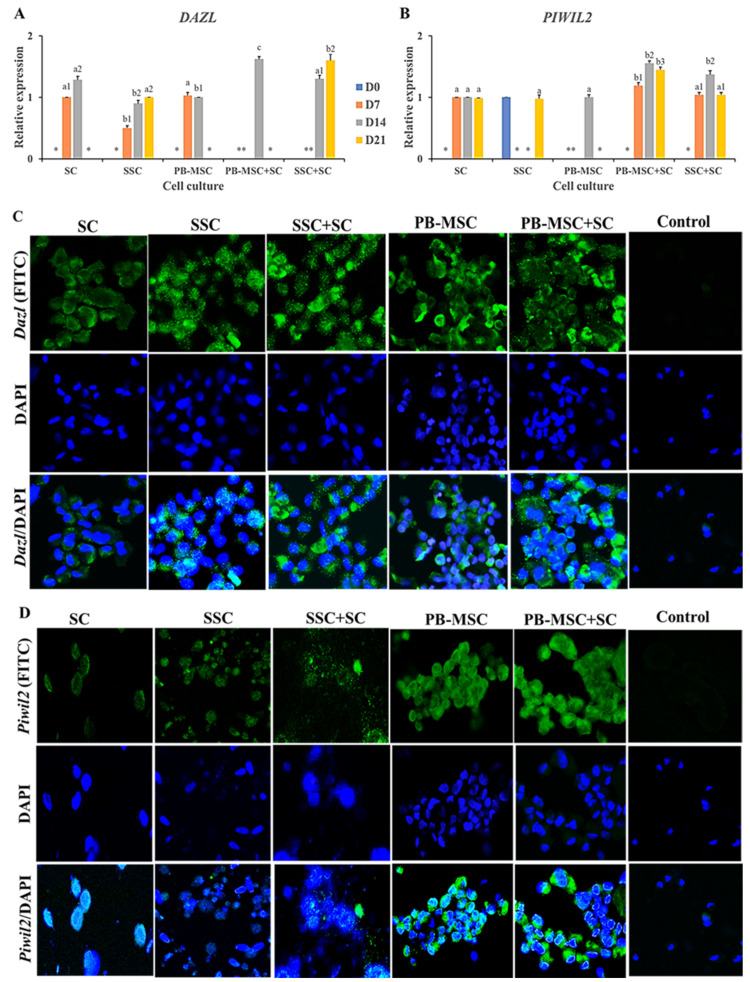
Expression of GC markers *DAZL* and *PIWIL2* in single cultures and co-cultures of bull PB-MSC, SSC with SC in 3D SACS for 21 days. (**A**) MSC+SC co-culture up-regulated (*p* < 0.05) *DAZL* gene expression on day 14, while SSC+SC co-culture up-regulated (*p* < 0.05) *DAZL* gene expression on day 21. (**B**) *PIWIL2* relative expression was higher (*p* < 0.05) in MSC+SC and SSC+SC co-cultures at day 14. At day 21 the relative expression of *PIWIL2* was higher (*p* < 0.05) in MSC+SC co-culture. (**C**) Intense immunofluorescence associated to *DAZL* was observed in SSC and PB-MSC single cultures and SSC+SC and MSC+SC co-cultures at day 14. (**D**) *PIWIL2* was immunodetected in PB-MSC+SC and SSC+SC co-cultures at day 14. (*) Indicate gene expression was not detected in a single sampling day. Different superscripts (a, b, c) indicate differences (*p* < 0.05) for the same marker between cell types and (1,2) for the same marker between culture days.

Co-culture of PB-MSC+SC in 3D SACS up-regulated (*p* < 0.05) gene expression of *DAZL* (1.6 ± 0.04-fold MSC) compared to SC and PB-MSC single cultures on day 14 (Figure 4A, Table 2). SSC+SC co-cultures increased (*p* < 0.05) *DAZL* gene expression on day 21 (1.6 ± 0.13-fold SSC) compared to SSC (0.9 ± 0.05-fold MSC). *DAZL* gene expression was not detected in SC single cultures. Accordingly, immunofluorescence associated to *DAZL* was intense in PB-MSC+SC and SSC+SC co-cultures; however, *DAZL* was also immunodetected in SC, SSC and PB-MSC single cultures at day 14 (Figure 4C). Levels of mRNA of *PIWIL2* were higher (*p* < 0.05) in PB-MSC+SC (1.6 ± 0.05-fold SC) and SSC+SC (1.4 ± 0.04-fold SC) co-cultures in days 7, 14 and 21 compared with SC, SSC and PB-MSC (1.0 ± 0.03-fold SC) (Figure 4B, Table 2). In accordance with mRNA levels, immunofluorescence associated to *PIWIL2* was more intense in cocultures of PB-MSC+SC and SSC+SC compared to single cultures at day 14 (Figure 4D).

**Figure 5:**
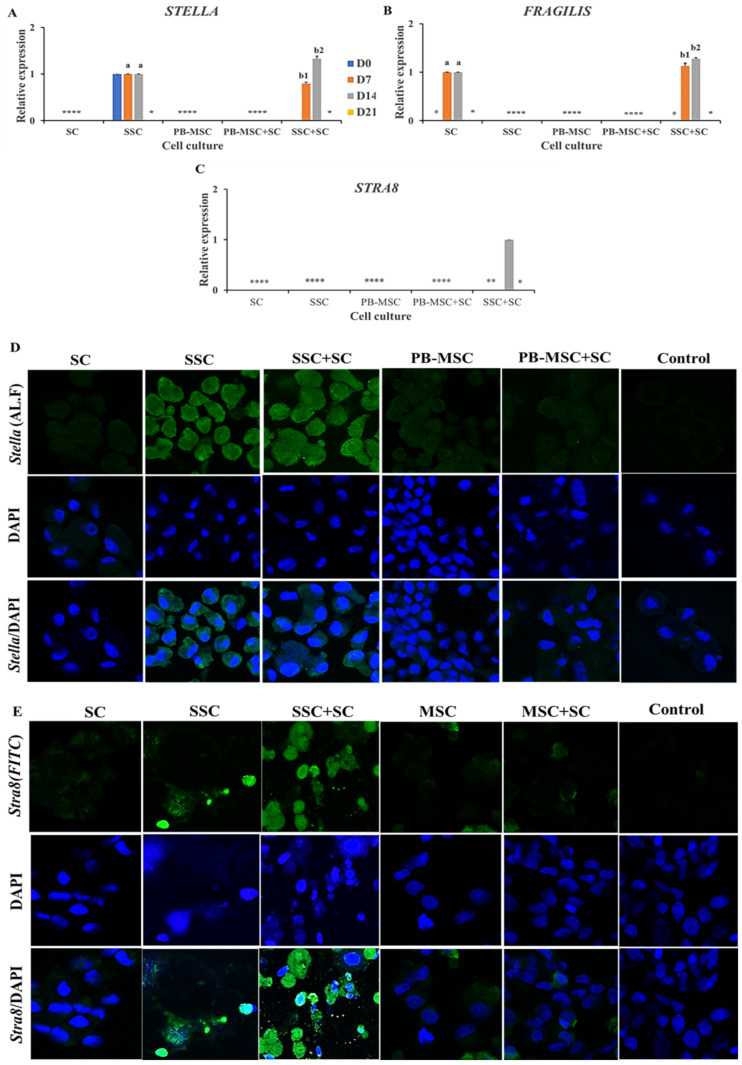
Expression of GC markers *STELLA*, *FRAGILIS* and *STRA8* in single cultures and co-cultures of bull PB-MSC, SSC with SC in 3D SACS for 21 days. (**A**–**C**) Higher expression of *STELLA* was detected on days 7 and 14 in co-cultures of SSC+SC compared to SSC. (**B**) Expression levels of *FRAGILIS* expression were higher (*p* < 0.05) in SSC+SC co-cultures compared to SC on days 7 and 14. (**C**) *STRA8* gene expression was only detected in SSC+SC co-culture on day 14. (**D**) Immunofluorescence associated to *STELLA* was observed in SSC and SSC+SC co-culture at day 14. (**E**) *STRA8* immunofluorescence was observed in SSC+SC co-culture at day 14. (*) Indicate gene expression was not detected in a single sampling day. Different superscripts (a, b) indicate differences (*p* < 0.05) for the same marker between cell types and (1,2) for the same marker between culture days.

Higher (*p* < 0.05) mRNA levels of *STELLA* were detected on day 14 in co-cultures of SSC+SC (1.3 ± 0.04-fold SSC) compared to SSC (Figure 5A, Table 2). *STELLA* gene expression increased (*p* < 0.05) in SSC+SC co-culture from day 7 to 14 day (0.8 ± 0.03-fold SC to 1.3 ± 0.04-fold SSC). Immunoreactivity for *STELLA* was detected in SSC and co-cultures of SSC+SC at day 14 of the experimental period (Figure 5D). Levels of *FRAGILIS* gene expression were higher (*p* < 0.05) in SSC+SC co-cultures (1.3 ± 0.03; 1.1 ± 0.05-fold SC) compared to SC on days 7 and 14 (Figure 5B, Table 2). Moreover, *FRAGILIS* gene expression increased (*p* < 0.05) in SSC+SC co-culture from day 7 to 14 day (1.1 ± 0.05-fold SC to 1.3 ± 0.03-fold SC). *STRA8* gene expression was only detected in SSC+SC co-culture on day 14 (Figure 5B, Table 2). Additionally, immunofluorescence associated to *STRA8* was observed in SSC and SSC+SC co-cultures at day 14 with a cytoplasmatic and nuclear pattern (Figure 5E). Gene expressions of SCP3 and VASA were not detected in either PB-MSC or SSC single cultures or co-cultures with SC.

### 3.6. Gene Expression of MSC Markers in PB-MSC Co-Cultured with SC in 3D SACS for 21 Days

**Figure 6:**
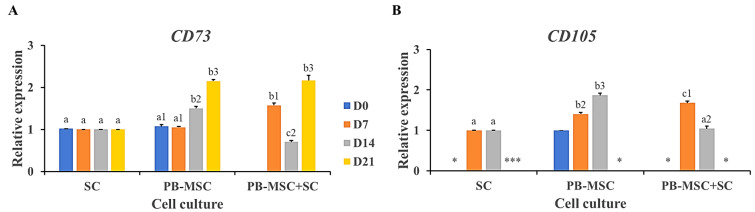
Expression of MSC markers *CD73* and *CD105* in single cultures and co-cultures of bull PB-MSC with SC in 3D SACS for 21 days. (**A**) MSC+SC co-culture down-regulated (*p* < 0.05) *CD73* gene expression on day 14. (**B**) *CD105* gene expression was down-regulated (*p* < 0.05) in the PB-MSC+SC on day 14 compared to PB-MSC. *CD105* gene expression was not detected in any culture at day 21. (*) Indicate gene expression was not detected in a single sampling day. Different superscripts (a, b, c) indicate differences (*p* < 0.05) for the same marker between cell types and (1,2,3) for the same marker between days of culture.

The mesenchymal cell-specific profile was evaluated in PB-MSC and SC single cultures and PB-MSC+SC co-cultures in 3D SACS during a 21-day experimental period. PB-MSC+SC co-cultures down-regulated (*p* < 0.05) gene expression of *CD73* and *CD105* (0.7 ± 0.02-fold SC, 1.0 ± 0.03-fold SC) on day 14 compared to PB-MSC and SC single cultures (1.5 ± 0.04-fold SC, 1.9 ± 0.04-fold SC) (Figure 6A,B, Table 2). In comparison, *CD73* gene expression increased (*p* < 0.05) in PB-MSC+SC co-cultures at day 7 (1.6 ± 0.04) and 21 (2.1 ± 0.02) of culture, compared to single cultures. *CD105* mRNA levels increased (*p* < 0.05) in PB-MSC+SC co-cultures at day 7 (1.9 ± 0.05-fold MSC), whereas *CD105* gene expression was not detected in PB-MSC+SC co-cultures at day 21.

## 4. Discussion

In the present study, we compared the potential for GC differentiation of bull PB-MSC and SSC in 3D SACS co-culture systems with SC during a 21-day culture period. Previous studies have reported difficulties in the isolation of PB-MSC due to limited availability in the circulating blood, diverse marker expression profiles and presence of hematopoietic cell precursors in the isolates [10]. In our study we used conventional Percoll separation for selection and standard culture system for expansion of bull PB-MSC, which resulted in obtention of adequate yields of PB-MSC for subsequent differentiation experiments. Furthermore, bull PB-MSC shared characteristics of bovine fetal BM-MSC and AT-MSC including fibroblastic morphology, high levels of expression of MSC markers *CD73* and *CD105,* and low levels of expression of hematopoietic molecules *CD45* and *CD34* [4,16,19]. Despite that previous studies on human [9,31] and ovine [32] cells have detected high levels of expression of CD90 in PB-MSC, one of the few previous analyses on bovine PB-MSC (7) did not analyze the expression of CD90. The role of CD90 in MSC biology is not clear; however, previous studies have indicated that reduction of CD90 expression resulted in increased potential for osteogenic and adipogenic differentiation of MSC, which suggests that CD90 may constitute an obstacle for MSC differentiation [33]. Overall, our analyses and previous data suggest that bull PB-MSC may display a different profile of CD90 expression compared to other species, and we can also speculate that the lack of CD90 expression may facilitate differentiation of bull PB-MSC. Despite the trilineage (osteogenic, adipogenic and chondrogenic) differentiation capacity of human PB-MSC have been previously reported [34], further studies are required to explore the mesodermal differentiation potential of bull PB-MSC. Nevertheless, PB-MSC represent an interesting source of MSC with various comparative advantages to other sources including availability of donors from different ages and sexes, avoidance of ethical concerns, minimally invasive collection procedures, and collection of adequate yields of MSC with specific profiles.

Cultures of SC used in this study were isolated from abattoir-derived testis and expanded as previously reported [19]. These SC populations expressed high mRNA levels of *WT1* and *AR* and displayed intense immunofluorescence associated to *WT1*. These results correspond to a previous study reporting a positivity of around 90% to *WT1* using FACS analysis [19]. Both *WT1* and *AR* markers have been previously identified as specific for SC in murine and human models [26,35]. *WT1* is related to the development of the gonad and acts as a transcription factor that regulates the inductive signal of the mesenchymal towards the coelomic epithelium of the mesonephros controlling the growth of the gonadal ridges that give rise to SC [36]. *AR* functions as a nuclear receptor and as a ligand-dependent transcription factor, which regulates the expression of a wide variety of genes involved in the development of puberty and male fertility [36]. Although SC populations used in the present study were isolated from testis by non-specific methods including enzymatic digestion and plastic adherence, these results indicate that homogeneous cultures of bull SC were obtained and used for subsequent differentiation experiments.

Bull SSC were isolated by two-step enzymatic digestion, sequential filtration and were separated by differential adhesion following a previously reported protocol [27]. The SSC population isolated through these methods expressed high mRNA levels of *UCHL1* and *CD90* and were immunopositive for *UCHL1.* Despite the lack of specific markers reported for SSC in domestic animals [37], *UCHL1* has been found to be consistently and specifically expressed in bull SSC [37,38,39]. *UCHL1* is related to the events of self-renewal and colocalization of SSC and is expressed in vivo in SSC located in the basement membrane of the seminiferous tubules, but not in differentiated GC [40,41]. *UCHL1* is expressed in male premeiotic GC and shows no affinity for somatic cells, making it an optimal marker for spermatogonia in domestic bull testes [37]. Moreover, *CD90* positive cells have been shown to exhibit cardinal properties of SSC, including proliferation, differentiation and colony formation in human and mouse models [31,42]. Our results indicate that SSC isolated from bull testes by enzymatic digestion, sequential filtration and differential adhesion display a consistent and homogeneous marker expression profile during subculturing. Furthermore, previous reports have indicated that putative SSC derived from domestic animals can be easily isolated and cultured; however, they suffer a gradual decline in proliferation during subculture, and, over time, spontaneous differentiation and apoptosis dominate cellular events, with the ultimate detention of SSC proliferation [37,43]. In this context, expansion of SSC under in vitro conditions could only be maintained for a short time, no more than 60 days, as a consequence of spontaneous differentiation to unspecific cell lineage [28,38,44,45]. In our study, however, primary cultures of SSC were kept in culture for intervals not exceeding 30 days, and, during this time, SSC maintained proliferation and were adherent with irregular and cuboidal morphology. Moreover, these bull SSC were able to respond to co-cultures with SC suggesting the absence of spontaneous differentiation.

In vitro co-cultures of PB-MSC+SC and SSC+SC in 3D SACS were established to recreate the testicular microenvironment and cell interactions with the aim to induce comparable patterns of differentiation in both bull PB-MSC and SSC into male GC. Cultures on 3D SACS can mimic tissue or organ-like characteristics and replicate the cellular environment in a more efficient manner compared to 2D cell cultures. As a result, 3D SACS may allow induced expression of the GC-specific profile of markers in a closer manner to those observed in vivo [29]. In the present study, 3D SACS induced formation of round and dense colonies in co-cultures of SSC+SC and MSC+SC from day 14 to 21, compared to more flat colonies in their single cultures of SC, PB-MSC and SSC. Similar changes in cell morphology have been reported in a co-culture of human SSC+SC for 14 days using 1% gelatin plates [46,47]. Formation of these round colonies may be the consequence of the 3D spatial distribution of cells and may also be associated to a tubular structure formation of seminiferous tubules with tight junctions. This structure may provide a better environment for interaction by cell–cell contact and for the paracrine secretion of SC.

Despite co-culture of PB-MSC with SC resulting in down-regulation of *NANOG* during the 21-day culture period, this co-culture induced up-regulation of *OCT4* at day 7. In comparison, co-culture of SSC with SC resulted in consistent expression of *NANOG*, *OCT4* and *SOX2* at day 14. *NANOG*, *OCT4* and *SOX2* are transcription factors that regulate common genes that promote pluripotency and self-renewal downstream, while inhibiting differentiation processes [48]. *NANOG* expression is promoted by *OCT*4 and *SOX2* expression [49,50], and together these three factors regulate a network of genes involved in the control of factors associated to chromatin remodeling, cell cycle and signal suppression [51]. These genes are expressed in both pluripotent GC and other early GC [51,52]. Consequently, its high expression indicates a state of undifferentiation in pluripotent cells and its down-regulation would occur during cell differentiation [46,52]. Thus, our results indicate that co-culture with SC in 3D SACS may induce different patterns of pluripotency marker expression representing different states of pluripotency in bull PB-MSC or SSC. While converse expression of *NANOG* and *OCT4* in PB-MSC+SC may indicate a partial induction of the differentiation process, consistent expression of *NANOG*, *OCT4* and *SOX2* in the SSC+SC co-cultures at day 14 may suggest the establishment of an undifferentiated or pluripotency state.

During co-culture in 3D SACS, SSC+SC increased the expression levels of *DAZL*, *PIWIL2*, *FRAGILIS* and *STELLA*, and activated the expression of *STRA8*. In comparison, co-culture of PB-MSC+SC only increased the expression levels of *DAZL* and *PIWIL2*. The results indicate that SSC+SC co-culture display a more robust GC differentiation profile characterized by the expression of more GC genes, compared with MSC+SC co-cultured under the same conditions. It is important to highlight that SSC are unipotent stem cells predetermined to sperm differentiation [53]. In comparison, PB-MSC are multipotent cells initially predetermined to differentiation into mesodermal lineages [2,3,54]. *DAZL* is essential for mammalian spermatogenesis and is involved in proliferation, development, maturation and functional maintenance of male GC [55]. The marker *PIWIL2* is expressed exclusively in GC, where it has a role in SSC self-renewal and spermatogenesis [56,57]. In the mouse, *PIWIL2* null mutants have been shown to have incomplete spermatogenesis and cannot produce spermatozoa [56]. Whereas *FRAGILIS*, a member of the interferon-induced family of transmembrane protein genes [58], is expressed in the genital crest at day 9.5 dais (days after intercourse) and then in the fetal gonad at day 13.5 [59]. The expression of *STELLA* is positive in the primordial GC during their migration to the dorsal mesentery at 8.5 dais, maintaining its expression up to 15.5 dais [58]. Bull PB-MSC displayed a more restricted GC profile compared to SSC, which suggests that co-culture with SC in 3D SACS represents a suitable culture system to compare GC differentiation of both PB-MSC and SSC. In addition, as indicated above, the more restricted GC profile of PB-MSC may be associated to its multipotentiality toward mesenchymal lineages including osteogenic, adipogenic and chondrogenic, compared to the unipotentiality of SSC toward GC and sperm. Previous studies have reported bovine AT-MSC and BM-MSC co-cultured with SC in a 2D system up-regulate *DAZL* and *PIWIL2* expression [19]. Moreover, exposure of bovine AT-MSC to BMP4 or RA for 14 days have also resulted in up-regulation of *PIWIL2* expression [16,17]. Overall, these results indicate that bovine MSC collected from different tissue sources may display a similar response after stimulation with different GC differentiation strategies, suggesting the presence of similar potential of MSC for GC differentiation but also the limited capacity of these culture systems to further induce advancement in the differentiation process.

Co-culture of PB-MSC+SC in 3D SACS for 21 days results in down-regulation of *CD73* and *CD105* gene expression at day 14. Similarly, our previous studies have reported down-regulation of *CD73* and CD105 in AT-MSC and BM-MSC co-cultured with SC in 2D system at day 14 [19]. *CD73* is considered a marker of MSC, is co-expressed with integrin β2 molecules and is involved in mechanisms of cell adhesion [54]. *CD105* is a receptor for the growth factors TGFβ1 and TGFβ3 and modulates its signaling by regulating the composition of different components of the extracellular matrix, including fibronectin and collagen [52,60]. Our results also showed that *CD73* and *CD105* were also expressed in bull SC, therefore they would not be selective for the PB-MSC population. Up-regulation expression of *CD73* mRNA on day 21 in PB-MSC+SC co-culture may be associated with the increased expression in single cultures of PB-MSC at the same day. However, down-regulation of *CD73* and *CD105* expression levels in the MSC+SC co-cultures on day 14 suggests that MSC would be losing the mesenchymal phenotype as an effect of SC interaction and progressing in the GC differentiation process.

## 5. Conclusions

Bull PB-MSC display some of the pre-defined requirements for human MSC, including adherence to plastic dishes under standard culture conditions, high expression of *CD73* and *CD105* and low expression of *CD45* and *CD34*. Co-culture of bull PB-MSC+SC and SSC+SC in 3D SACS results in differential expression of pluripotency and GC markers. Bull SSC display a more robust GC differentiation profile compared to PB-MSC, which may be associated to their unipotency toward sperm differentiation. Based on the patterns of expression of pluripotency and GC markers detected in SSC+SC and MSC+SC co-cultures, we can conclude that day 14 constitutes the period when SSC and MSC co-cultured with SC partially reached a stage of early GC.

## Figures and Tables

**Table 1:** Sequence of primers used for Q-PCR analysis.

Gene	Nucleotide Sequence (5′-3′)		Access Number
	Forward	Reverse	
Housekeeping genes			
*β-ACTINA*	CGCACCACTGGCATTGTCAT	TCCAAGGCGACGTAGCAGAG	NM_173979.3
*GAPDH*	CCTTCATTGACCTTCACTACATGGTCTA	TGGAAGATGGTGATGGCCTTTCCATTG	NM_001034034.2
Mesenchymal stem cell genes			
*CD73*	TGGTCCAGGCCTATGCTTTTG	GGGATGCTGCTGTTGAGAAGAA	NM_174129.3
*CD105*	CGGACAGTGACCGTGAAGTTG	TGTTGTGGTTGGCCTCGATTA	NM_00107639.1
Hematopoietic cell genes			
*CD34*	CATGCCGTCTTAACCCATCT	CGGTCTACAGAGGTGGTGGT	NM_174009.1
*CD45*	CCACGGGTATTCAGCAAGTT	CCCAGATCATCCTCCAGAAA	NM_001206523
Sertoli cell genes			
*WT1*	CGTGCGTACCATGTAGGGAA	CTCGTGCTTGAAGGAGTGGT	XM_015474834.2
*AR*	CAGATGGCAGTCATTCAG	CTTGGTGAGCTGGTAGAAG	XM_001244127
Spermatogonial stem cell genes			
*UCHL1*	AGAAGCAGCATCTCGGTTCC	CGTGGTTGAGGGTAAGTGCT	NM_001046172.2
*CD90*	ACTCATACCGCTCCCGAACCA	CATGTGTATGTCCCCTCGTCCTT	NM_001034765
Germ cell genes			
*DAZL*	TCCAAGTTCACCAGTTCAGG	CGT CTG TAT GCT TCT GTC CAC	NM_001081725.1
*STRA8*	TGTGCCCAGGTGTTCATCTC	GGGGACTGTCACCTCATTGG	XM_015463130
*PIWIL2*	TCGTATTGATGATGTGGATTGG	GGGAGCAGCAGGATTTCAC	XM_617223.3
*FRAGILIS*	ATCTGCAGCGAGACCTCTGT	CCGATGGACATGATGATGAG	XM_002697323
*STELLA*	TGCAAGTTGCCACTCAACTC	TCTTACCCCTCTCCGCCTAT	NM_00111110
*SCP3*	CTAGAATTGTTCAGAGCCAGAG	GTTCAAGTTCTTTCTTCAAAG	NM_001040588.2
*VASA*	TGCTACTCCTGGAAGACTGA	CGGTCTGCTGAACATCTCTA	NM_001007819.1
Pluripotency genes			
*OCT4*	GAAAGAGAAAGCGGACGAG	GTGAAAGGAGACCCAGCAG	NM_174580.2
*NANOG*	TAAGCACAGGGGGCAAAAGT	ATGGCTAAAAGGGGTGGAGG	NM_001025344.1
*SOX2*	CCCGTGGTTACCTCTTCTTCC	CGCTCTGCTAGTGCTGGGAC	NM_001105463.2

**Table 2:** Gene expression pattern of mesenchymal, pluripotency, GC and male GC genes in bull co-cultures of PB-MSC and SSC with SC in 3D SACS at day 14 compared to single cultures of MSC, SC and SSC.

Gene	PB-MSC+SC	SSC+SC
*CD73*	↓ (Q-PCR)	-
*CD105*	↓ (Q-PCR)	-
*DAZL*	↑ (Q-PCR) *(IF)	↑(Q-PCR) *(IF)
*STRA8*	x (Q-PCR)	+ (Q-PCR) *(IF)
*PIWIL2*	↑ (Q-PCR) *(IF)	↑ (Q-PCR) *(IF)
*FRAGILIS*	x (Q-PCR)	< (Q-PCR)
*STELLA*	x (Q-PCR)	< (Q-PCR) *(IF)
*OCT4*	x (Q-PCR) *(IF)	→ (Q-PCR) *(IF)
*NANOG*	x (Q-PCR)	→ (Q-PCR) *(IF)
*SOX2*	x (Q-PCR)	+ (Q-PCR)
*SCP3*	x (Q-PCR)	x (Q-PCR)
*VASA*	x (Q-PCR)	x (Q-PCR)

Abbreviations: **+**, Activated expression; →, No change in expression; ↑, Increased expression; ↓, Decreased expression; **x**, No expression detected; **-** Not evaluated; *, Immunodetected; Q-PCR, Quantitative PCR; IF, Immunofluorescence.

## Data Availability

The data presented in this study are available on request from the corresponding author.
